# The Anti-apoptosis Effect of Single Electroacupuncture Treatment *via* Suppressing Neuronal Autophagy in the Acute Stage of Ischemic Stroke Without Infarct Alleviation

**DOI:** 10.3389/fncel.2021.633280

**Published:** 2021-02-02

**Authors:** Ying Xing, Min Zhang, Man-Man Wang, Ya-Shuo Feng, Fang Dong, Feng Zhang

**Affiliations:** ^1^Department of Rehabilitation Medicine, The Third Hospital of Hebei Medical University, Shijiazhuang, China; ^2^Department of Pathophysiology, Hebei Medical University, Shijiazhuang, China; ^3^Department of Clinical Laboratory Medicine, The Third Hospital of Hebei Medical University, Shijiazhuang, China; ^4^Hebei Key Laboratory of Critical Disease Mechanism and Intervention, Shijiazhuang, China

**Keywords:** stroke, autophagy, apoptosis, electroacupuncture, SIRT1, JNK

## Abstract

The main purpose of the study was to investigate the antiapoptotic effect of electroacupuncture (EA) in the acute stage of ischaemic stroke in rats. The cerebral ischemia model was established by middle cerebral artery occlusion (MCAO)/reperfusion in rats. A single EA treatment was performed at the acute stage of ischaemic stroke. The neurological function, brain water content, apoptotic cell number, and cerebral infarct volume were assessed in stroke rats. The expression of autophagy-related proteins (LC3II/I, Beclin1, P62, and LAMP1), Sirtuin 1 (SIRT1), p-JNK, p-ERK1/2, and cleaved caspase-3 (CCAS3) were measured by Western blot, immunofluorescence, and immunohistochemistry. Rapamycin (RAP, an activator of autophagy) was used to confirm the antiapoptotic effect of EA *via* regulating autophagy. The brain edema infarct size and apoptotic cell number were increasing within 3 days following stroke, and brain edema reached its peak at 24 h after stroke. EA treatment at 24 h after ischaemic stroke obviously suppressed the number of apoptotic cells and brain edema. However, there were no significant differences in infarct volumes among EA-12 h, EA-24 h, and MCAO/R group. Moreover, EA treatment at 24 h after ischaemic stroke obviously suppressed the expression of CCAS3, LC3II/I, Beclin1 while increasing the level of P62 and LAMP1 and hence mediating autophagy, which was reversed by RAP. Meanwhile, the expression of SIRT1, p-ERK1/2, p-JNK were promoted by EA at 24 h after ischaemic stroke. In conclusion, EA treatment may suppress apoptosis possibly *via* regulating autophagy in the acute period after ischaemic stroke, hence reducing brain injury.

## Introduction

Stroke is a common disease that causes disability and death worldwide. The occurrence of acute first-ever ischaemic stroke is significantly higher than acute first-ever hemorrhagic and other types of stroke, according to the Global Burden of Disease (GBD) 2015 study (Roth et al., 2017). Ischaemic stroke is a fatal disease that is capable of resulting in neurological defects due to the occlusion of blood flow to certain brain areas (He et al., 2018). Recombinant tissue-type plasminogen activator (rt-PA) treatment has been well established for ischaemic stroke therapy, but only a few ischaemic stroke patients can obtain benefits from rt-PA treatment because of the restrained therapeutic time window (Shi et al., 2015). Therefore, effective and safe therapy is urgently required in a clinical setting.

Electroacupuncture (EA) is a modified technique based on traditional acupuncture therapy; EA stimulates selected acupoints *via* an electrical current, replacing manual manipulations and bringing more reproducible outcomes both in clinical settings and the laboratory (Liu et al., 2019). EA has been widely adopted as a rehabilitation method for stroke patients in clinical settings, which can alleviate neurological dysfunction without obvious adverse effects (Kim et al., 2018). According to the meridians theory of Traditional Chinese Medicine (TCM), spasticity and flaccid paralysis after stroke are two different performances involving in an imbalance of “liver qi,” and the acupoints of the Stomach and Large Intestines, such as Zusanli and Quchi, could be used for rebalancing the “liver qi” (Chang et al., 2020; Zou et al., 2020). The EA at acupoints of Quchi and Zusanli is commonly applied in stroke (Zou et al., 2020). In our previous studies, we demonstrated that EA at Quchi and Zusanli acupoints might exert an anti-apoptosis effect by activating the MAPK pathway (Xing et al., 2018). However, whether EA can regulate neuronal autophagy levels to alleviate apoptosis remains unclear.

Autophagy is a catabolic process that occurs with the formation of autophagolysosome, which acts as removing impaired cellular and molecular components (Moreira et al., 2017). Autophagy may be activated in various conditions, including hypoxic stimulus, oxidative stress, starvation, and nonfunctional protein accumulation (Zhang and Chen, 2018). To the best of our knowledge, excessive activation of autophagy may facilitate neuronal death *via* precipitating neurocytes to self-digest their own constituents or by interacting with the apoptotic cascade around the ischaemic areas following ischaemic stroke (Liu et al., 2016). EA might improve cerebral ischemia/reperfusion (I/R) injury by suppressing excessive autophagy during the reperfusion period, which might be a vital cause of EA protecting neurocytes (Ting et al., 2017).

Sirtuin 1 (SIRT1) is a nicotine adenine dinucleotide (NAD^+^)-dependent class III histone deacetylase and expresses in all cell types, and SIRT1 is associated with the alleviation of hippocampus ischemia/reperfusion injury, which is involved in neuronal autophagy and apoptosis (Zhou et al., 2018). The activation of SIRT1 plays a crucial role in maintaining redox balance and mitochondrial function and suppressing apoptosis *via* activating the MAPK signaling pathway (Becatti et al., 2018). Similar to ERKs, JNKs are a subfamily of MAPKs and are richly expressed in the central nervous system. Furthermore, the regulation of the JNK pathway has been proven to play a neuroprotective role after ischaemic stroke (Xing et al., 2018). Interestingly, the inhibition of the JNK pathway is also involved in mitigating autophagy and reducing neuronal apoptosis (Xue et al., 2017). Therefore, the JNK pathway might be a downstream pathway of SIRT1 when it comes to taking part in the regulation of autophagy.

In the current study, we explore the effect of EA treatment in the early stage of I/R and the detailed mechanism of EA in inhibiting apoptosis by regulating autophagy possibly through the SIRT1-JNK and ERK pathway.

## Materials and Methods

### Experimental Animals

All experimental animal protocols were approved by the Animal Care and Use Committee of Hebei Medical University, and all the procedures were conducted according to the recommendations of the Laboratory Animal—Guide for Ethical Review of Animal Welfare (GB/T35892–2018). A total of male Sprague–Dawley rats (*n* = 284, 230–270 g) were purchased from the Laboratory Animal Centre of Hebei Medical University, Hebei, China.

### Inclusion and Exclusion Criteria

A neurological behavior score assessment was used to determine the inclusion criteria of rats with I/R. Rats whose score was between 1 and 3 were included. All rats were randomly allocated to different groups by computer-generated randomization schedules. For drug experiments, the rats were randomly allocated to groups before drug administration, and rats with scores between 1 and 3 were included. The details are shown in Supplementary Table 1.

### Animals’ Groupings

**Figure 1:**
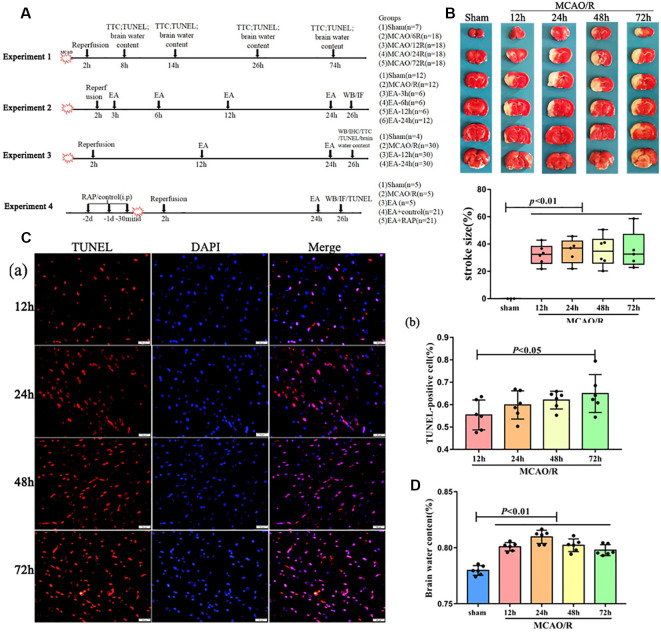
The pathology damage after ischemia/reperfusion (I/R) within 3 days. **(A)** Experimental groups and the protocol. **(B)** 2,3,5-Triphenyltetrazolium Chloride (TTC) staining and the statistical analysis showing cerebral infarct volume of the rats. **(Ca)** Terminal deoxynucleotidyl transferase dUTP nick-end labeling (TUNEL)staining showing several apoptotic cells in the penumbra of the infarct area of rats. **(Cb)** Bar diagram showing the number of apoptotic cells. **(D)** Bar diagram showing the percentage of the water content of the brain.

The animal groupings are shown in Figure 1A. The details are shown in the Supplementary Material.

### Drug Injection

Rapamycin (RAP, 3 mg/kg/day, Selleck Chemicals, United States) or control (2% DMSO, 30% PEG, 5% Tween 80) was injected intraperitoneally (i.p) for three consecutive days, and the last injection was conducted 30 min before the Middle cerebral artery occlusion (MCAO) surgery.

### Middle Cerebral Artery Occlusion (MCAO)

Male Sprague–Dawley rats (250–280 g) were housed in the same condition during a 12 h light/dark cycle and used to establish the MCAO/reperfusion model, as previously described. In short, the rats were anesthetized *via* intraperitoneal injection of 3% sodium pentobarbital (50 mg/kg) and fixed on the operating table. The rectal temperature of the rats was kept at 37 ± 0.5°C during the whole procedure *via* a heating pad. Subsequently, the right common carotid artery (CCA), internal carotid artery (ICA), and external carotid artery (ECA) were isolated; then CCA and ECA were ligated with a thread. The origin of the middle cerebral artery was occluded for 2 h with a nylon suture, which was inserted from the left ECA into the ICA. Afterward, the suture was withdrawn for blood reperfusion. The sham group underwent the same procedure without the insertion of the nylon suture. Finally, the rats returned to their cages and were permitted free access to water and food following recovery from anesthesia. Successful occlusions (<20% baseline) and reperfusion were confirmed by Laser-doppler flowmetry.

### Neurological Behaviors Assessment

The neurological deficits score was used in all rats 2 h after reperfusion in the blind method, as described previously (Longa et al., 1989). The details are shown in the Supplementary Material.

### Cerebral Edema Measurements

The brain tissues were immediately weighed to measure the wet weight (WW) and then were placed in a drying oven at 70°C. After drying for 72 h, brain tissues were measured again to obtain the dry weight (DW). A percentage of brain water content was calculated by the following formula: (WW − DW)/WW×100%.

### Electroacupuncture Stimulation

EA treatment was performed at the acupoints of Quchi (LI11) and Zusanli (ST36) in the right affected limb using an apparatus (Model G6805–2A; Shanghai Huayi Company, Shanghai, China). The specific methods were as per a previous method (Xing et al., 2018). The stimulation (disperse-dense waves of 4/20 Hz, 4 V) lasted for 30 min.

### TTC (2,3,5-Triphenyltetrazolium Chloride) Staining

The infarct size was measured as described. Six rats in each group were sacrificed, and the fresh brains were quickly obtained and then immediately frozen at −20°C for 20 min. Then, the brain tissues were sectioned coronally into six slices (2-mm/section) for TTC staining. The slices were stained in 2% TTC at 37°C for 30 min. Finally, the stained slices were photographed with a Canon camera, and the infarct size percentage (%) was calculated using ImageJ.

### TUNEL Staining

Terminal deoxynucleotidyl transferase dUTP nick-end labeling (TUNEL) staining was used to assess neuronal apoptosis using the *in situ* Cell Death Detection Kit (Roche, Mannheim, Germany). The brains were treated with 4% formaldehyde at 4°C for 24 h, followed by embedding in paraffin. Subsequently, those brains were cut into 5-um-thick coronal slices. Afterward, brain slices were treated with proteinase K for 15 min and quenched with 3% H_2_O_2_ for 10 min at the door temperature. Following incubating in the TUNEL reaction mixture for 60 min, the cell nuclei were stained by DAPI, and red fluorescence of TUNEL-positive cells (apoptotic cells) was assessed with a light microscope (Olympus Corporation, Tokyo, Japan). The five high-power fields of the ischaemic penumbra were randomly selected. The number of apoptotic cells was counted by a blinded observer using the ImageJ software.

### Western Blot

The expression levels of Beclin-1, LC3, P62, LAMP1, p-JNK, cleaved caspase-3 (CCAS3), SIRT1, and p-ERK1/2 in the peri-ischaemic hippocampus in each group were analyzed by Western blot. The rats in each group were sacrificed at different times following reperfusion. Subsequently, the peri-ischaemic hippocampus was quickly dissected and then lysised in the RIPA lysis buffer. BCA kits were used to measure protein concentration. Equal amounts of proteins (30 μg) from each sample were subjected to 12% or 15% SDS-PAGE and then transferred to PVDF membranes. Afterward, the PVDF membranes were blocked for 1 h at 37°C. We then prepare the primary antibodies: anti-Beclin1 (1:2,000; Abcam), anti-LC3 (1:1,000; Cell Signal Technology), anti-P62 (1:2,000; Proteintech), anti-LAMP1 (1:1,000; Abcam), anti-p-ERK1/2 (1:1,000; Cell Signal Technology), anti-SIRT1 (1:1,000; Abcam) and CCAS3 (1:300; Proteintech). After incubation with the diluted primary antibodies at 4°C overnight, the PVDF membranes were treated with horseradish peroxidase-conjugated secondary antibodies (1:5,000) for 2 h at room temperature and immunodetected by an enhanced chemiluminescent substrate. The protein expression level was normalized to the GAPDH (1:5,000; Proteintech) protein expression. The band density was analyzed with ImageJ software (1.46r).

### Immunofluorescence (IF) and Immunohistochemistry (IHC) Staining

Twenty-four hours following reperfusion, the rats were sacrificed for IF staining. The brains were removed and immediately fixed with PBS containing 4% paraformaldehyde. After 24 h of fixation, the brains were dehydrated, tansparented, and then embedded into paraffin blocks. Paraffin blocks were sectioned into coronal slices of 5 μm thickness. The sections were treated with a blocking solution at 37°C for 30 min. The slices were incubated with an anti-LC3 antibody (1:200, Cell Signal Technology) at 4°C overnight. After washing in PBS for 5 min three times, a secondary antibody was then incubated at 37°C for 30 min. After washing in PBS (pH 7.4) for 15 min, the slices were incubated with DAPI at room temperature for 10 min. IHC was performed by anti-CCAS3 antibody (1:100, Cell Signal Technology) incubation and BDA, staining followed by dehydration. In all sections, the hippocampus fields were selected for photography and the expression levels of the LC3 and CCAS3 proteins were calculated in this area. The average fluorescence intensity and positive cell number were analyzed with the ImageJ software to evaluate the protein expression level in all groups.

### Statistical Analysis

Statistical analyses were implemented using SPSS 25.0 software (IBM, Armonk, NY, USA). The data from multiple groups were analyzed using a one-way analysis of variance (ANOVA) followed by a least significant difference (LSD) test. All data are presented as mean ± $\bar{X}$. Statistical significance was set at *p* < 0.05.

## Results

### The Pathological Changes After I/R Within 3 Days

As shown in Figure 1, to detect the injury of cerebral I/R, the rats at different reperfusion times were euthanized and tested with TTC staining, TUNEL staining, and assessment of brain water content. Our results showed that the brain infarct volume and the number of apoptotic cells were increasingly elevated by TTC and TUNEL staining with reperfusion time (Figures 1B,C). However, the brain water content of the rats was significantly increased within 3 days after surgery, especially at 24 h (*p* < 0.01; Figure 1D).

### The Effect of EA Treatment on Autophagy in Ischaemic Stroke Outset

**Figure 2:**
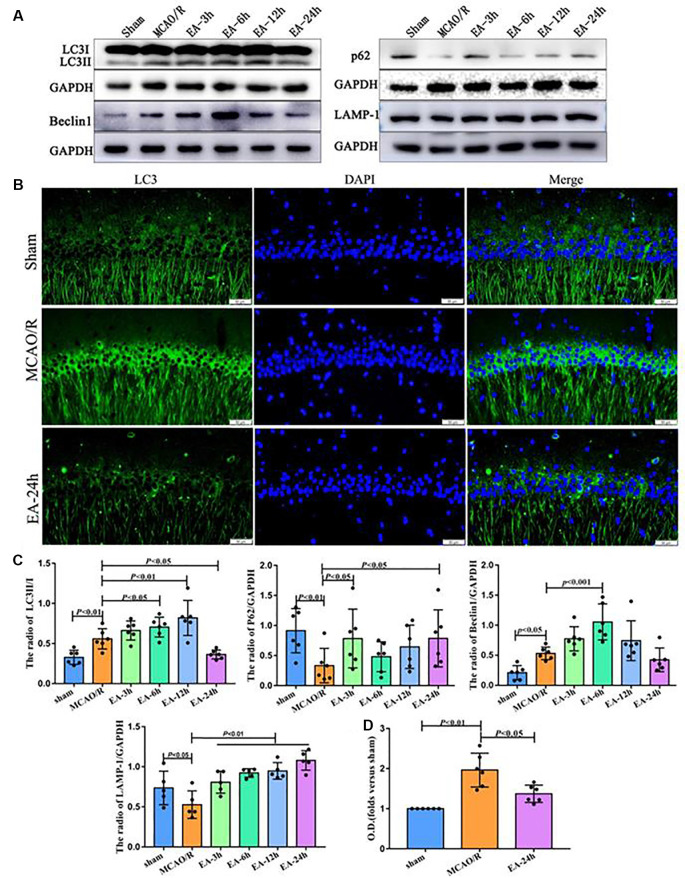
The effect of electroacupuncture (EA) treatment on autophagy in ischaemic stroke outset. **(A)** Western blot analysis showing the difference of LC3II/I, P62, Beclin1, and LAMP-1 after EA treatment at 3, 6, 12, and 24 h *post*-middle cerebral artery occlusion (MCAO) surgery. **(B)** Immunofluorescence (IF) analysis showing the expression difference of LC3 after EA treatment among the sham, MCAO/R, and EA-24 h groups. **(C)** Statistical analysis of the expression of autophagy-related proteins assessed by Western blot. **(D)** Statistical analysis of the LC3 expression assessed by IF. The control images of GAPDH are re-used for illustrative purposes in Figure 2.

We assessed the levels of autophagy-related proteins (LC3, P62, LAMP1, and Beclin1) after a single EA treatment at 3, 6, 12, and 24 h following MCAO surgery, to clarify the speculation that EA may regulate the autophagy level. As shown in Figures 2A,C, the expression of LC3II/I and Beclin1 increased in the MCAO/R group than the sham group (*p* < 0.01). Compared with the MCAO/R group, LC3II/I was significantly increased in the EA-6 h (*p* < 0.05) and EA-12 h group (*p* < 0.01) and Beclin1 was significantly increased in the EA-6 h group (*p* < 0.001), but LC3II/I (*p* < 0.05) was significantly decreased in the EA-24 h group. Also, the expressions of P62 (*p* < 0.01) and LAMP1 (*p* < 0.05) were decreased in the MCAO/R group compared with the sham group. However, the expressions of P62 (*p* < 0.05) and LAMP1 (*p* < 0.01) in the EA-24 h group were increased compared with the MCAO/R group. LC3 levels found with immunofluorescence were significantly increased in the MCAO/R group (*p* < 0.01) and decreased in the EA-24 h group (*p* < 0.05; Figures 2B,D). These results demonstrate that EA treatment at 24 h after surgery may inhibit autophagy, which might be related to the neuroprotection mechanism.

### The Neuroprotective Effect of EA Treatment in Ischaemic Stroke Outset

**Figure 3:**
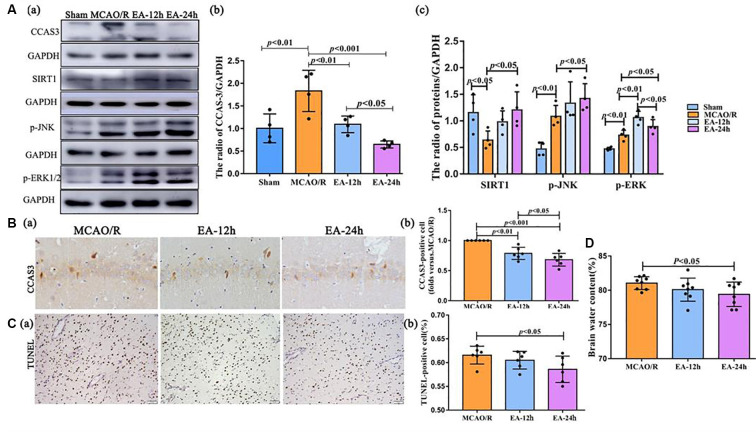
The neuroprotective effect of EA treatment in ischaemic stroke outset. **(Aa–c)** The Western blot and bar diagram shows the expression of cleaved caspase-3 (CCAS3), Sirtuin 1 (SIRT1), p-JNK, and p-ERK1/2 after EA treatment. **(Ba,b)** Immunohistochemistry (IHC) and bar diagram show the number of CCAS3 positive cells in the ipsilateral hippocampus of the rats. **(Ca,b)** TUNEL staining and bar diagram shows the apoptotic cell number in the penumbra of the infarct area of the rats. **(D)** Bar diagram shows the percentage of brain water content. The control images of GAPDH are re-used for illustrative purposes in Figure 3.

Since the autophagy level had an obvious change after EA intervention at post-stroke 12 and 24 h, as shown in Figure 3, we next explored the neuroprotective effect of EA at 12 and 24 h after surgery. The TUNEL staining, brain water content assessment, and TTC staining were performed to clarify the neuroprotection of EA against I/R injury. The number of apoptotic cells was obviously reduced in the EA-24 h group than the MCAO/R group (*p* < 0.05; Figure 3C). The brain edema was significantly alleviated in the EA-24 h group than the MCAO/R group (*p* < 0.05; Figure 3D). However, there was no obvious difference in infarct volume among MCAO/R, EA-12 h, and EA-24 h group (*p* > 0.05), as shown in Supplementary Figure 1.

Western blot and IHC were performed to assess the level of CCAS3. This was done because CCAS-3 plays a control role in the phase of apoptosis. As shown in Figure 3A, CCAS3 levels were significantly increased in the MCAO/R group than the sham (sham surgery) group (*p* < 0.01). As shown in Figures 3A,B, CCAS3 was significantly decreased in the EA-24 h and EA-12 h group compared with the MCAO/R group, and the expression of CCAS3 in the EA-24 h group had a more obvious decrease than the EA-12 h group (*p* < 0.05). These results demonstrate that EA treatment may effectively decrease the apoptosis level at 24 h after stroke.

Also, the levels of SIRT1 were significantly decreased in the MCAO/R group compared with the sham group (*p* < 0.05), but the EA treatment reversed this change at 24 h after MCAO surgery (*p* < 0.05). The p-JNK levels were significantly increased in the MCAO/R group than in the sham group (*p* < 0.01) and were further increased in the EA-24 h group than the MCAO/R group (*p* < 0.05). Furthermore, the p-ERK1/2 levels were significantly increased in the MCAO/R group than the sham group (*p* < 0.01) and were further increased in the EA-12 h (*p* < 0.01) and EA-24 h (*p* < 0.05) groups than the MCAO/R group; also, the expression of p-ERK1/2 in the EA-12 h group was higher than the EA-24 h group (*p* < 0.05; Figure 3A). Thereby, we speculate that the antiapoptotic effect of EA might be associated with the SIRT1/JNK pathway and the ERK1/2 pathway.

### Rapamycin (Autophagy Activator) Aggravated Apoptosis After EA Treatment at 24 h After MCAO

**Figure 4:**
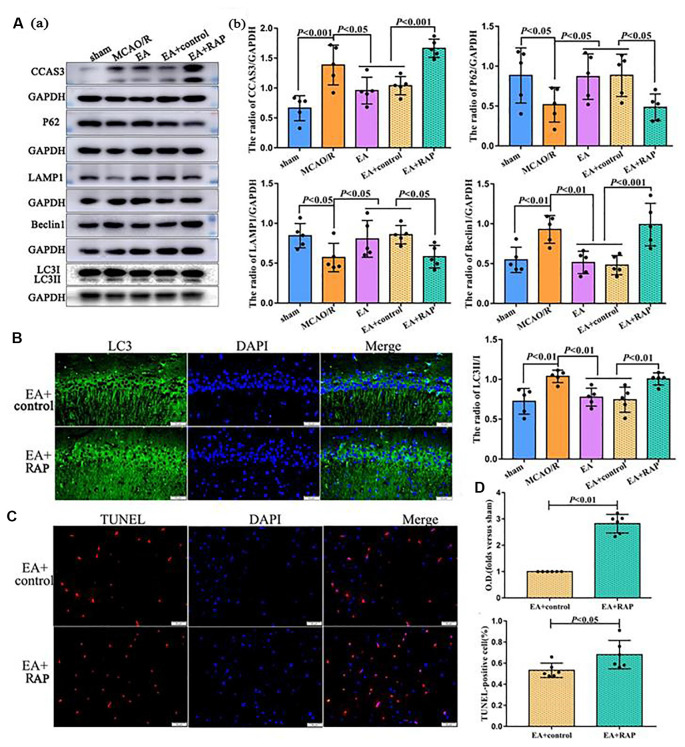
Rapamycin (RAP, autophagy activator) aggravated apoptosis after EA treatment at 24 h after MCAO. **(Aa)** Western blot showing the level of CCAS3 and autophagy-related proteins in the ipsilateral hippocampus of the rats. **(Ab)** Statistical analysis of protein levels as assessed by Western blot. **(B)** IF showing the LC3 levels in the ipsilateral hippocampus of the rats. **(C)** TUNEL staining showing the apoptotic cell levels in the penumbra of the infarct area of rats. **(D)** Statistical analysis of the LC3 levels as assessed by IF staining and the number of apoptotic cells as assessed by TUNEL staining. The control images of GAPDH are re-used for illustrative purposes in Figure 4.

Finally, we explored the role of autophagy in the process of EA regulating neuronal apoptosis through RAP administration. As shown in Figures 4Aa,b, the CCAS3 (*p* < 0.001), LC3-II/I (*p* < 0.01), and Beclin1 (*p* < 0.01) levels were significantly increased, and P62 (*p* < 0.05) and LAMP1 (*p* < 0.05) levels decreased in the MCAO/R group compared with the sham group (*p* < 0.01). EA treatment may reverse the expression of these proteins. After RAP (autophagy activator) treatment, CCAS3 (*p* < 0.001), LC3II/1 (*p* < 0.01), and Beclin1 (*p* < 0.001) levels in the EA + RAP group were significantly increased than the EA and EA+control groups. Moreover, the expressions of P62 and LAMP1 in the EA + RAP group were lower than the EA and EA+control groups (*p* < 0.05). There was no difference in CCAS3, LC3-II/I, Beclin1, P62, and LAMP1 expression between the EA and EA+control groups (*p* > 0.05). The LC3 level as analyzed by immunofluorescence was also significantly increased in the EA + RAP group compared with the EA +control group (*p* < 0.01; Figures 4B,D). The number of apoptotic cells in the EA + RAP group was significantly increased compared with the EA+control group (*p* < 0.05; Figures 4C,D).

## Discussion

In this study, we provide new insights into the detailed mechanism for the antiapoptotic effect of EA and its ability to suppress neuronal autophagy in the early stage of ischaemic stroke, which might be associated with the SIRT1-JNK and ERK pathway. EA treatment is a potential strategy for promoting patient recovery in the acute stage of ischaemic stroke. Autophagy and its capacity to regulate apoptosis might be a potential therapeutic target for exploring new therapies for ischaemic stroke in the future.

Previous studies have demonstrated that brain edema occurs immediately after cerebral infarction, and the most obvious promotion of cerebral edema has been observed at 24 h after reperfusion (Hu et al., 2014), which is as per our results. Also, as shown in our results, the cerebral infarct area and the number of apoptotic cells in the penumbra of the infarct area increased with time over a period of 3 days following I/R injury. Therefore, initiating early treatment through an effective and safe method is a rational therapeutic strategy to alleviate I/R injury.

EA is a technique that integrates electric stimulation and acupuncture to stimulate specific acupoints, creating more reproducible outcomes compared with acupuncture alone (Liu et al., 2019). EA is involved in regulating various pathological mechanisms pre and post-stroke, including apoptosis, autophagy, and inflammation, and so on (Wang et al., 2011; Shu et al., 2016). A single EA treatment in the acute stage of ischaemic stroke also may alleviate apoptosis, inflammation, and oxidative stress by activating the parasympathetic nervous system (Chi et al., 2018). As shown in our previous studies, EA treatment can significantly exert an antiapoptotic effect after cerebral I/R (Xing et al., 2018). However, the concrete mechanism for the antiapoptotic effect of EA in stroke remains unclear.

In a normal physiological state, the autophagy level in cells is always low; however, it may be significantly activated in response to nutritional deficiency (Sato et al., 2019), oxidative stress (Sun et al., 2019), and immune response (Padhi et al., 2019). Extensive and excessive autophagy may damage cellular components and ultimately cause cell death (Zhao et al., 2010). EA may regulate autophagy activity (Ting et al., 2017), which might be associated with various signaling pathways, including PI3K-AMPK and mTOR pathways (Codogno and Meijer, 2005). In the current study, we found that the expression level of P62 and LAMP1 were significantly increased in the hippocampus at 24 h compared to the MCAO/R group after stroke, and the LC3-II/I ratio was significantly increased at 12 h after MCAO surgery and was decreased at 24 h after MCAO surgery in the hippocampus after EA treatment; this result might be involved in the neuroprotective effect of EA and post-stroke 12 h and 24 h might be the key time points for stroke intervention methods application.

In the subsequent experiments, we found that even a single EA treatment could exert neuroprotective effects *via* reducing the percentage of brain edema and the numbers of apoptotic cells, especially at 24 h following ischemic stroke, which was associated with an antiapoptotic effect. We further confirmed the relationship between autophagy and apoptosis by RAP. The activation of autophagy by RAP reversed the antiapoptotic effect of EA, indicating that the antiapoptotic effect of EA is significantly regulated by the autophagy level.

SIRT1, one of the Sirtuin protein family members, is reported to play a crucial role in the neuroprotective effect against ischaemic injury, which is involved the regulation of autophagy and apoptosis (Zhou et al., 2018). The JNK pathway plays different roles in the early and late stages of cerebral ischaemic stroke (Murata et al., 2012). Also, ERK1/2 has been reported as being involved in EA pretreatment-inhibited apoptosis *via* cannabinoid receptor type 1 (Du et al., 2010). In our study, the expression level of SIRT1 was inhibited after I/R injury, and the expression level of p-ERK and p-JNK was activated following I/R injury. EA intervention markedly promoted the expression of SIRT1, p-ERK, and p-JNK and suppressed neuronal autophagy and apoptosis, thus alleviating brain damage following I/R. Thus, SIRT-1, ERK, and JNK might involve in regulating apoptosis and autophagy after EA treatment in the acute stage of ischaemic stroke.

## Conclusion

Taken together, the present results demonstrate that EA exerts neuroprotection against cell apoptosis *via* regulating neuronal autophagy following ischaemic stroke. The current study provides novel insights that possibly SIRT1, JNK, and ERK1/2 might be potential therapeutic targets to alleviate neuronal apoptosis after ischaemic stroke. In summary, EA treatment might be a rational strategy for promoting patient recovery in the acute stage of ischaemic stroke, thus benefiting the increasing number of stroke patients worldwide.

## Data Availability Statement

The raw data supporting the conclusions of this article will be made available by the authors, without undue reservation.

## Ethics Statement

The animal study was reviewed and approved by Animal Care and Use Committee of Hebei Medical University.

## Author Contributions

YX and FZ designed the study. YX, MZ, Y-SF, M-MW and FD performed the experiments. FD, M-MW and FZ analyzed the results together. YX and FZ wrote the article. All authors contributed to the article and approved the submitted version.

## Conflict of Interest

The authors declare that the research was conducted in the absence of any commercial or financial relationships that could be construed as a potential conflict of interest.
