# Supplementary material for: The Anti-apoptosis Effect of Single Electroacupuncture Treatment via Suppressing Neuronal Autophagy in the Acute Stage of Ischemic Stroke Without Infarct Alleviation
Source: Front Cell Neurosci. 2021 Feb 2;15:633280. doi: 10.3389/fncel.2021.633280 (PMC7884854; doi:10.3389/fncel.2021.633280)
Supplement: Supplementary file 1 [file Data_Sheet_1.DOCX]

### The anti-apoptosis effect of Electroacupuncture via suppressing neuronal autophagy in acute stage of ischemic stroke

### Figures:1, Tables:1

**Methods**

#### Inclusion and exclusion criteria

A neurological behaviour score assessment was used to determine the inclusion criteria of rats with I/R. Rats whose score was between 1 and 3 were included. All rats were randomly allocated to different groups by computer-generated randomisation schedules. For drug experiments, the rats were randomly allocated to groups before drug administration, and rats with scores between 1 and 3 were included. The details are shown in Table I in the online-only dada supplement.

**Animals’ groupings**

Experiment 1: The degree of brain damage was compared at 24 h, 48 h and 72 h following reperfusion.

Seventy-nine rats were randomly divided into five groups: (1) sham group (n=7): rats underwent an incision of skin without the occlusion of the middle cerebral artery; (2) MCAO/12R group (n=18): all rats underwent surgery. Observation was performed at 12 h after reperfusion; (3) MCAO/24R group (n=18): all rats underwent surgery. Observation was performed at 24 h after reperfusion; (4) MCAO/48R group (n=18): all rats underwent surgery. Observation was performed at 48 h after reperfusion; (5) MCAO/72R group (n=18): all rats underwent surgery. Observation was performed at 72 h after reperfusion.

The results indicated that the ischaemic rats showed significant brain oedema at 24 h post-reperfusion compared with those at 12h, 48h, 72h after surgery. The apoptotic cell number and infarct volume increased with reperfusion time within 3 days. Accordingly, the time points within 24 h after stroke were chosen to explore the best treatment time after stroke outset.

Experiment 2: EA regulated autophagy level within 24 h after MCAO surgery.

To detect the influence of different times of EA stimulation on the autophagy level within 24 h after ischaemic stroke, we divided 54 rats into six groups: (1) sham group(n=12); (2) MCAO/R(n=12); (3) EA-3h(n=6): EA stimulation performed at 3 h after MCAO surgery;(4) EA-6h(n=6): EA stimulation performed at 6 h after MCAO surgery; (5) EA-12h(n=6): EA stimulation were performed at 12 h after MCAO surgery; and (6) EA-24h(n=12): EA stimulation were performed at 24 h after MCAO surgery.

Six rats in each group were euthanatized, and the hippocampus of rats were isolated to detect the protein levels using Western blot analysis. In sham, MCAO/R and EA-24h groups, other six rats were used for an immunofluorescence analysis. The results indicated that EA treatment at 12h and 24 h after surgery had significant effect on autophagy. Thereby, we speculate that EA intervention at 12h or 24h after MCAO surgery might exert neuroprotection against I/R injury.

Experiment 3: Neuroprotectioneffect of EA stimulation at 12 h or 24 h following MCAO surgery.

Nighty-four rats were randomly divided into four groups: (1) sham group (n=4); (2) MCAO/R group (n=30): the rats were administered the same as the MCAO/24R group; (3) EA-12h group (n=30); (4) EA-24h group (n=30).

The results indicated that EA treatment at 24 h after surgery had the best neuroprotective effect by suppressiong cell apoptosis. Accordingly, we believed that 24 h after surgery would be the best treatment time for stroke. Next, we explored the antiapoptotic mechanism of EA at 24 h after surgery.

Experiment 4: autophagy involved in the antiapoptotic effect of EA.

Fifty-seven rats were randomly divided into five groups: (1) sham group (n=5); (2) MCAO/R group (n=5); (3) EA group (n=5); (4) EA+control group (n=21): a control was provided to the rats by [intraperitoneal](file:///C:\Program%20Files%20(x86)\Application\7.5.2.0\resultui\dict\?keyword=intraperitoneal)[injection](file:///C:\Program%20Files%20(x86)\Application\7.5.2.0\resultui\dict\?keyword=injection)(i.p) 3 days before surgery; (5) EA+RAP group(n=21): rapamycin was provided to the rats by [i](file:///C:\Program%20Files%20(x86)\Application\7.5.2.0\resultui\dict\?keyword=intracerebroventricular).p 3 days before surgery.

**Neurological behaviours assessment**

The neurological deficits score was used in all rats 2 h after reperfusion using the blind method, as described previously(Zhang et al., 2019). The specific method is as follows: grade 0, no neurologic dysfunction symptoms; grade 1, flexion of the contralateral front jaw when a rat was picked up by the tail; grade 2, circling to the contralateral side when walking; grade 3, serve rotating to the contralateral side when walking; grade 4, not able to walk. Rats with 1–3 points indicated successful establishment of the MCAO model.

**References**

Al-Ahmady, Z.S., et al.,2019. Selective Liposomal Transport through Blood Brain Barrier Disruption in Ischemic Stroke Reveals Two Distinct Therapeutic Opportunities. ACS Nano. <http://dx.doi.org/> 10.1021/acsnano.9b01808.

Chen, W.C., et al.,2019. Aryl hydrocarbon receptor modulates stroke-induced astrogliosis and neurogenesis in the adult mouse brain. J Neuroinflammation 16**,** 187. <http://dx.doi.org/> 10.1186/s12974-019-1572-7.

Lai, T.K.Y., et al.,2019. The receptor-receptor interaction between mGluR1 receptor and NMDA receptor: a potential therapeutic target for protection against ischemic stroke. FASEB J fj201900417R. <http://dx.doi.org/> 10.1096/fj.201900417R.

LoPresti, S.T., et al.,2019. Free radical-decellularized tissue promotes enhanced antioxidant and anti-inflammatory macrophage response. Biomaterials 222**,** 119376. <http://dx.doi.org/> 10.1016/j.biomaterials.2019.119376.

Shen, P., et al.,2017. Cortical spreading depression preconditioning mediates neuroprotection against ischemic stroke by inducing AMP-activated protein kinase-dependent autophagy in a rat cerebral ischemic/reperfusion injury model. J Neurochem 140**,** 799-813. <http://dx.doi.org/> 10.1111/jnc.13922.

Velasquez, D.A., et al.,2011. The central Sirtuin 1/p53 pathway is essential for the orexigenic action of ghrelin. Diabetes 60**,** 1177-1185. <http://dx.doi.org/> 10.2337/db10-0802.

Wang, X., et al.,2014. Pre-ischemic treadmill training alleviates brain damage via GLT-1-mediated signal pathway after ischemic stroke in rats. Neuroscience 274**,** 393-402. <http://dx.doi.org/> 10.1016/j.neuroscience.2014.05.053.

Wu, M., et al.,2018. Rapamycin prevents cerebral stroke by modulating apoptosis and autophagy in penumbra in rats. Ann Clin Transl Neurol 5**,** 138-146. <http://dx.doi.org/> 10.1002/acn3.507.

Xing, Y., et al.,2018. Electroacupuncture Alleviated Neuronal Apoptosis Following Ischemic Stroke in Rats via Midkine and ERK/JNK/p38 Signaling Pathway. J Mol Neurosci 66**,** 26-36. <http://dx.doi.org/> 10.1007/s12031-018-1142-y.

Zhang, C.Y., et al.,2019. Nanoparticle-induced neutrophil apoptosis increases survival in sepsis and alleviates neurological damage in stroke. Sci Adv 5**,** eaax7964. <http://dx.doi.org/> 10.1126/sciadv.aax7964.

**Supplementary Table 1**

| **Experiment 1** | | | | | | | | | | | | | | | | | | | |
| --- | --- | --- | --- | --- | --- | --- | --- | --- | --- | --- | --- | --- | --- | --- | --- | --- | --- | --- | --- |
|  | **sham** | | | | | **MCAO/12R** | | | | **MCAO/24R** | | | | **MCAO/48R** | | | **MCAO/72R** | | |
| **n** | 7 | | | | | 18 | | | | 18 | | | | 18 | | | 18 | | |
| **Score** | 0 | | | | | 2.06±0.64 | | | | 1.93±0.83 | | | | 1.91±0.75 | | | 1.89±0.76 | | |
| **Experiment 2** | | | | | | | | | | | | | | | | | | | |
|  | | | **sham** | | **MCAO/R** | | | | **EA-3h** | | | **EA-6h** | | | **EA-12h** | | | | **EA-24h** |
| **n** | | | 12 | | 12 | | | | 6 | | | 6 | | | 6 | | | | 12 |
| **Score** | | | 0 | | 1.83±0.72 | | | | 2.17±0.75 | | | 1.83±0.41 | | | 2.00±0.63 | | | | 1.92±0.80 |
| **Experiment 3** | | | | | | | | | | | | | | | | | | | |
|  | | | | **sham** | | | | **MCAO/R** | | | | | **EA-12h** | | | **EA-24h** | | | |
| **n** | | | | 4 | | | | 30 | | | | | 30 | | | 30 | | | |
| **Score** | | | | 0 | | | | 1.93±0.83 | | | | | 1.91±0.75 | | | 1.89±0.76 | | | |
| **Experiment 4** | | | | | | | | | | | | | | | | | | | |
|  | | **sham** | | | | | **MCAO/R** | | | | **EA** | | | **EA+control** | | | | **EA+RAP** | |
| **n** | | 5 | | | | | 5 | | | | 5 | | | 21 | | | | 21 | |
| **Score** | | 0 | | | | | 2.00±0.71 | | | | 2.00±0.82 | | | 1.95±0.86 | | | | 1.86±0.65 | |

**Supplementary Fig 1**

**
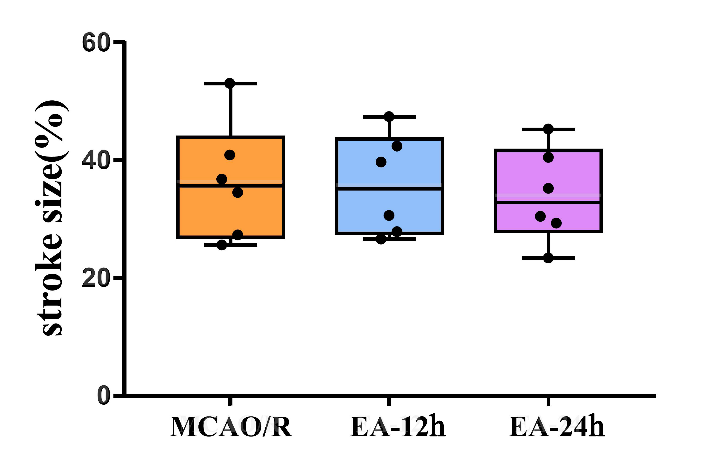
**

**Supplementary Fig 1** The infarct volume among EA-12h, EA-24h and MCAO/R group. There were not significant differences of infarct volumes among EA-12h, EA-24h and MCAO/R group.
