# Supplementary figures and images for: The Anti-apoptosis Effect of Single Electroacupuncture Treatment via Suppressing Neuronal Autophagy in the Acute Stage of Ischemic Stroke Without Infarct Alleviation
Source: Front Cell Neurosci. 2021 Feb 2;15:633280. doi: 10.3389/fncel.2021.633280 (PMC7884854; doi:10.3389/fncel.2021.633280)

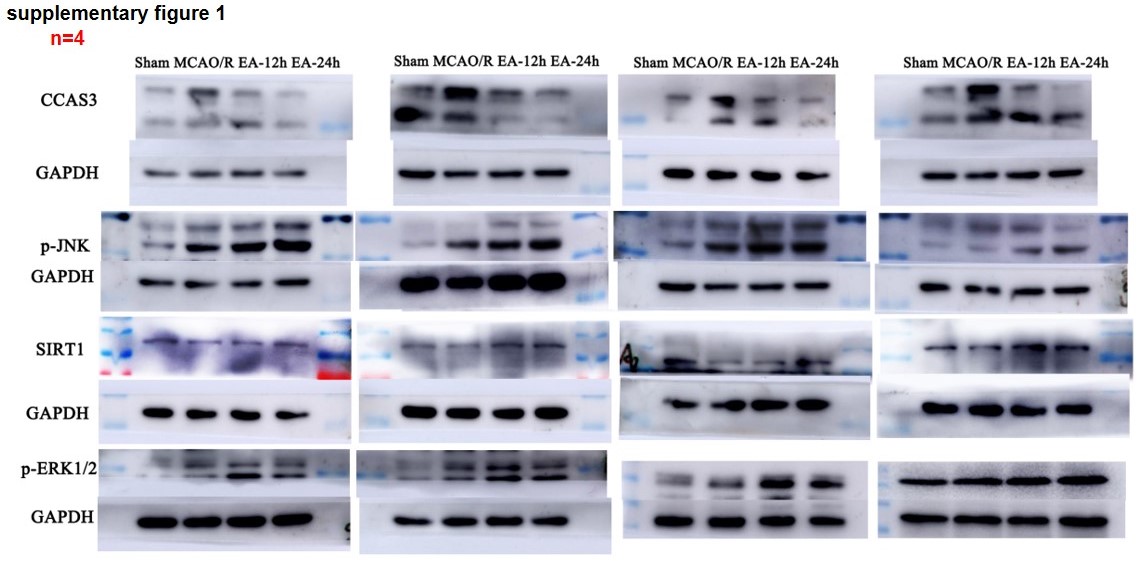

Supplement: Supplementary file 2 [file Image_1.JPEG]

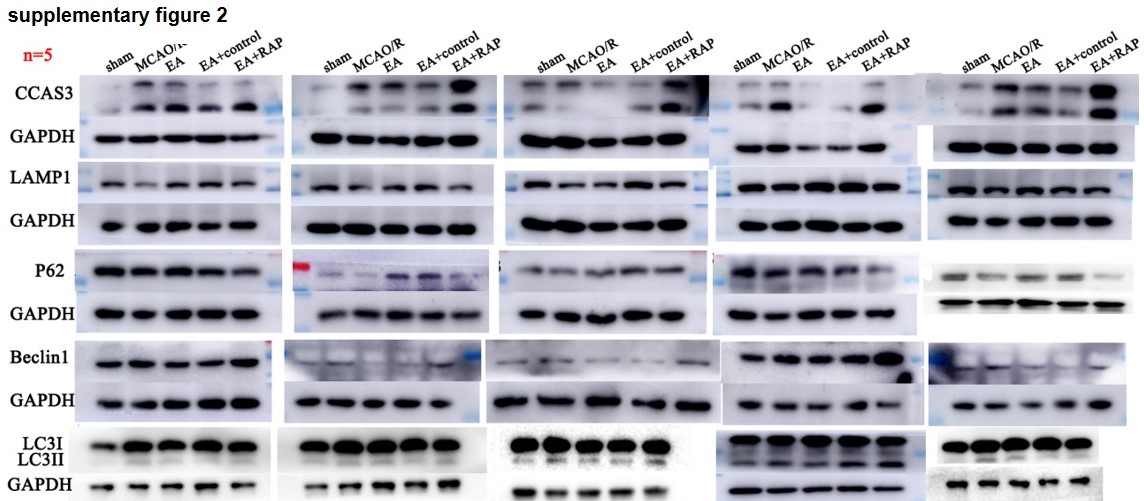

Supplement: Supplementary file 3 [file Image_2.JPEG]
